# Many phish in the C: A coexisting‐choice‐criteria model of security behavior

**DOI:** 10.1111/risa.13947

**Published:** 2022-05-14

**Authors:** Iain Embrey, Kim Kaivanto

**Affiliations:** ^1^ Glen Urquhart High School Drumnadrochit UK; ^2^ Department of Economics Lancaster University Lancaster UK

**Keywords:** advanced persistent threat, choice criteria, dual‐process theory, latent class model, phishing, peripheral‐route persuasion, states of mind, social engineering

## Abstract

Normative decision theory proves inadequate for modeling human responses to the social‐engineering campaigns of advanced persistent threat (APT) attacks. Behavioral decision theory fares better, but still falls short of capturing social‐engineering attack vectors which operate through emotions and peripheral‐route persuasion. We introduce a generalized decision theory, under which any decision will be made according to one of multiple coexisting choice criteria. We denote the set of possible choice criteria by C. Thus, the proposed model reduces to conventional Expected Utility theory when $|C_{\text{EU}}|=1$, while Dual‐Process (thinking fast vs. thinking slow) decision making corresponds to a model with $|C_{\text{DP}}|=2$. We consider a more general case with |C|≥2, which necessitates careful consideration of *how*, for a particular choice‐task instance, one criterion comes to prevail over others. We operationalize this with a probability distribution that is conditional upon traits of the decisionmaker as well as upon the context and the framing of choice options. Whereas existing signal detection theory (SDT) models of phishing detection commingle the different peripheral‐route persuasion pathways, in the present descriptive generalization the different pathways are explicitly identified and represented. A number of implications follow immediately from this formulation, ranging from the conditional nature of security‐breach risk to delineation of the prerequisites for valid tests of security training. Moreover, the model explains the “stepping‐stone” penetration pattern of APT attacks, which has confounded modeling approaches based on normative rationality.

## 1 INTRODUCTION

The human element in decision making is not only deliberative, but also emotional, intuitive, and fallible. Social‐engineering campaigns target and exploit these nondeliberative features of human decision making (Cialdini, 2007; Hadnagy, 2011; Langenderfer & Shimp, 2001; Mitnick & Simon, 2002; Oliveira et al., 2017; Petty & Cacioppo, 1986; Rusch, 1999). A major lacuna for security‐behavior modeling is that standard decision theory fails to capture the peripheral‐route persuasion pathways that are exploited in social‐engineering campaigns.

In contrast, signal detection theory (SDT) has been successfully adapted to model human responses to phishing attacks.^1^ The flexibility of SDT is instrumental in this context. It has been used to study the descriptive value of normative decision theory, behavioral decision theory, and the combination of behavioral decision theory and susceptibility to peripheral‐route persuasion (Kaivanto, 2014). Unsurprisingly, the latter combination proves most useful and informative. Nevertheless, two limitations may be observed in the existing SDT‐based approach: (i) decisionmakers are assumed to be permanently characterized by one fixed decision‐making model, and (ii) the effects of different peripheral‐route persuasion pathways feed into, and become commingled in, a single value of the discriminability parameter.^2^ Descriptive validity favors relaxation of the former, while interpretability of modeling favors relaxation of the latter.

We introduce a generalization of decision theory that fulfills these desiderata.^3^ The generalization comprises two principal components.

First, a nondegenerate set C of “ways of deciding”—here called “choice criteria”—which in the phishing context includes not only subjective expected utility (SEU) to capture rational deliberative decision making, but also prospect theory (PT) which captures behavioral decision making (Tversky & Kahneman, 1992), a “routinely click‐straight‐through” element that captures unmotivated and unthinking routinized actions (automaticity) (Moors & DeHouwer, 2006), and an “impulsively click‐through” element that captures emotionally motivated impulsive actions (Cialdini, 2007; Hadnagy, 2011; Langenderfer & Shimp, 2001; Mitnick & Simon, 2002; Oliveira et al., 2017; Petty & Cacioppo, 1986; Rusch, 1999). This approach therefore generalizes not only SEU and PT, but also dual‐process (DP) theories.^4^

Our approach also formalizes the notion—to which the article's title alludes—that there are several distinct types or classes of phishing ploy, and that individuals' susceptibility differs across qualitatively distinct social‐engineering attack vectors. It is important to distinguish between these distinct phishing attack vectors—both to understand individuals' behavioral responses to them, and to understand organizations' total security‐breach risk exposure. A phishing ploy that plays upon the prospect of a time‐delimited opportunity for wealth is constructed very differently—and is processed very differently by its recipient(s)—than a phishing ploy that plays upon employees' standard routines of unquestioningly responding to bosses' and colleagues' emails, opening any appended email attachments, and clicking on enclosed links. An organization's email security training may effectively address the former, but in many organizations the latter remains a worrying vulnerability.

The second component of the generalization is a conditional probability distribution over the different choice criteria, that is, over the elements of the set C. As each new choice task is confronted, a draw from this distribution determines which choice criterion becomes operative, and so we will refer to it as the *State‐of‐Mind* (SoM) distribution for an individual *i* at time *t*. We allow an individual's SoM distribution to be conditional upon: their psychological traits and decision experiences, the situational context of the decision, and the framing of the choice options. This approach is similar to that of two existing addiction models (Bernheim & Rangel, 2004; Laibson, 2001) although we extend those models by allowing the framing of the choice options to be strategically determined by an adversarial agent (the attacker), and by allowing both the prior experiences and situational context of a decision to be strategically influenced by an allied agent (the Information Security Officer [ISO]).

A key advantage of the present formulation is the top‐level differentiation of the decisionmaker's susceptibility to different kinds of phishing ploys. This formulation yields a number of immediate implications. First, the overall security‐breach risk due to phishing cannot be conceived in unconditional terms. Since an individual's susceptibility to phishing depends on the type of phishing ploy, the phishing‐ploy‐*type* exposure distribution takes on importance, as does the intensity of this exposure (i.e., the total number of phishing emails traversing the spam filter) and the quality of phishing‐ploy execution. Second, a single test‐phishing email is insufficient for evaluating the effectiveness of email security training. email security training does not necessarily generalize across different choice criteria. Hence, a single test‐phishing email may determine the robustness of security practice toward one particular phishing ploy, but it is orthogonal to potential vulnerabilities within the remaining choice criteria. Third, not only is the organization's security‐breach risk conditional, but the attacker gets to *choose* the phishing‐ploy‐type exposure distribution, as well as the intensity of this exposure. The attacker has first‐mover advantage. Moreover, the attacker always has the option to develop *new* phishing‐ploy types that are not addressed by the organization's existing working practices and training materials. Fourth, given working practices in most organizations and given the dimensions over which the attacker can tailor a phishing campaign, it is clear that the attacker can attain a very high total probability of successfully breaching the target organization's cybersecurity. In part, this is due to the fact that typical working practices in nonhigh‐security organizations^5^ do not involve special treatment of embedded links or attached files.^6^ It is also due to the *disjunctive* accumulation (addition, rather than multiplication) of successful‐security‐breach probabilities over spam‐filter‐traversing phishing emails. But it is also due to the scope for using rich contextual information to tailor a campaign into a *spear‐phishing* attack—that is, to specifically target the “routinely click‐straight‐through” choice criterion characterized by automaticity.

Furthermore, our model supports an explanation for the “stepping‐stone penetration pattern” that is common in APT attacks.^7^ Whereas models of security behavior premised upon normative rationality have not been successful in explaining the stepping‐stone pattern, we show that in light of a coexisting‐choice‐criteria model of security behavior, the stepping‐stone penetration pattern may be recovered as a constrained‐optimal attack vector.

The sequel is organized as follows. Section 2 briefly reviews the phishing literature, showing that phishing attacks employ social‐engineering techniques that circumvent deliberatively rational decision processes. Section 3 reviews the empirical literature which has documented multiple “ways of deciding,” thereby establishing a rigorous empirically grounded basis for the coexisting‐choice‐criteria model. Section 4 introduces the coexisting‐choice‐criteria model, and illustrates some of its properties, including its ability to support an explanation of the stepping‐stone penetration pattern (Section 4.3). Section 5 presents the results of a randomized controlled experiment which provides evidence for the practical usefulness of our model. Section 6 summarizes the high‐level insights afforded by the coexisting‐choice‐criteria model and discusses its implications for understanding stepping‐stone attacks. This concluding section also discusses the model's implications for ISOs, emphasizing design and validation of antiphishing training as well as embedding security culture within broader organizational culture.

## 2 PHISHING TARGETS THE HUMAN ELEMENT

The capacity for rational deliberation is a feature of human beings, albeit not the overriding trait it was thought to be when Carl Linnaeus coined the binary nomenclature, *Homo sapiens*.^8^ Both large‐scale and narrowly targeted social engineering are predicated upon the intuitive, emotional, and fallible nature of human behavior, and it is now recognized that psychology is an essential component—alongside engineering and economics—for understanding information security (Anderson & Moore, 2009).

More than half of all US government network‐security‐incident reports concern phishing attacks, and the number of phishing emails being sent to users of federal networks is growing rapidly (Johnson, 2013; US OMB, 2012). The Federal Bureau of Investigation (FBI) and the Department of Homeland Security (DHS) recently issued an amber alert warning of APT activity targeting energy—especially, nuclear power^9^—and other sectors (FBI and DHS, 2017). In this broad APT campaign, spear phishing was the preferred initial‐breach technique. The corporate sector is targeted more widely, commonly using phishing to create an entry point, for the purposes of extortion, illegally acquiring customer‐information (and credentials) databases, as well as for acquiring commercially sensitive information. The incidence of corporate cyber espionage is not systematically disclosed, but many of the high‐profile examples of corporate hacking that have come into the public domain were staged via phishing (Elgin et al., 2012).

Online scams such as phishing and spear phishing employ techniques of persuasion that have collectively been labeled “social engineering” (Hadnagy, 2011; Rusch, 1999). These techniques eschew direct, rational argumentation in favor of “peripheral” routes to persuasion. The most prominent of these peripheral pathways to persuasion are, in no particular order: (i) authority, (ii) scarcity, (iii) similarity and identification, (iv) reciprocation, (v) consistency following commitment, and (vi) social proof (Cialdini, 2007; Hadnagy, 2011; Langenderfer & Shimp, 2001; Mitnick & Simon, 2002; Oliveira et al., 2017; Petty & Cacioppo, 1986; Rusch, 1999). Scams^10^ typically augment peripheral‐route persuasion by setting up a scenario that creates psychological pressure by triggering *visceral emotions* that override rational deliberation (Langenderfer & Shimp, 2001; Loewenstein, 1996, 2000). Visceral emotions—such as greed, envy, pity, lust, fear, and anxiety—generate psychological discomfort as long as the underlying need remains unfulfilled, and psychological pleasure or even euphoria when that need is fulfilled. The manipulative scenario is deliberately structured so that the scammer's proposition offers the double prospect of relief from the visceral discomfort as well as visceral satisfaction upon fulfilling the underlying need.

An ideally scripted scam scenario contrives a compelling, credible need for immediate action. If a scam‐scenario script falls short of this ideal, it will almost invariably emphasize the urgency with which action must be taken (Langenderfer & Shimp, 2001; Loewenstein, 1996, 2000). In itself, this introduces visceral anxiety where none existed before, and simultaneously, precludes the availability of time for cooling off and for rational deliberation. Visceral emotions have both a direct hedonic impact as well as an impact via altering the relative desirability of different cues and attributes. Crucially, visceral emotions also affect how decisionmakers process information, narrowing and restricting attention to the focal hedonic cue and its availability (or absence) in the present (Loewenstein, 1996, 2000). Since visceral emotions—and their concomitant effects on attention and relative desirability of different cues/attributes—are short‐lived, scam scripts contrive reasons for immediate action.^11^

At sufficiently high levels of intensity, visceral emotions can override rational deliberation entirely (Loewenstein, 1996). Mass phishing scams often aim to exploit human emotions in this fashion. Spear phishing attacks, on the other hand, typically aim to exploit the intuitive and fallible nature of human decision making without necessarily stoking emotion. This approach targets the routinization and *automaticity* (Moors & DeHouwer, 2006) upon which successful management of a high‐volume inbox rests. In psychology, automaticity is associated with features such as unintentionality, uncontrolled/uncontrollability, goal independence, purely stimulus‐driven action, unconscious action, and fast and efficient action (Moors & DeHouwer, 2006). For most civilian organizations outside the security community, employees trust emails—and any embedded URLs and file attachments—sent by bosses and immediate colleagues, and frequently also those sent by more distant contacts. Failure to do so would bring most organizations to a slow crawl. Spear phishing thus exploits this routine and unquestioning trust that is automatically extended to bosses, colleagues, and contacts—and unintendedly, to plausible facsimiles thereof.

More surprising is the fact that spear phishing emails endowed with rich contextual information have been deployed successfully on both sides of the civilian/noncivilian and security/nonsecurity divides. A partial list of successfully breached governmental, defense, corporate, and scientific organizations includes the White House, the Australian Government, the Reserve Bank of Australia, the Canadian Government, the Epsilon mailing list service, Gmail, Lockheed Martin, Oak Ridge National Laboratory, RSA SecureID, Coka Cola Co., Chesapeake Energy, and Wolf Creek Nuclear Operating Corporation (Elgin et al., 2012; Hong, 2012; Johnson, 2013; Perlroth, 2017; US OMB, 2012). When implemented well with appropriate contextual information, a spear‐phishing email simply does not attract critical evaluation, and its contents are acted upon in a routine or automatic fashion.

Contextual cues also feature centrally in models of end‐user response to phishing emails (Cranford et al., 2019) based on instance‐based learning theory (IBLT) (Gonzalez et al., 2003). The latter, which is a model of dynamic decision making in cognitive science, also draws on instance‐based learning algorithms (IBLAs) (Aha et al., 1991) from machine learning. IBLT has proven to be a tractable and useful framework for modeling phishing detection, providing insights, for example, into the effect of exposure frequency on phishing email detection (Singh et al., 2019). Natural language processing (NLP)‐based IBLAs offer up the possibility of scoring the emotional impact of a message upon its recipient—thereby opening an avenue to computational implementation of coexisting‐choice‐criteria models.^12^

## 3 COEXISTING CHOICE CRITERIA: EMPIRICAL PROVENANCE

Decision theorists are increasingly coming to terms with the implications of DP theory, which has been developed by psychologists and popularized by Daniel Kahneman (2012) in *Thinking, Fast and Slow*.

Meanwhile, a well‐established stream of empirical‐decision‐theory literature offers legitimation for the notion that there may be more than one way of reaching a decision. That literature captures heterogeneity in choice criteria with finite mixture (FM) models. Standard estimation procedures for such models allow the data to determine how many different choice criteria are present, and then to provide, for each individual, the respective criterion‐type membership probabilities.^13^ In Harrison and Rutström's FM models,^14^ the traditional single‐criterion specification is statistically rejected, in their words providing “a decent funeral for the representative agent model that assumes only one type of decision process” (Harrison & Rutström, 2009). In turn, Coller et al.'s (2012) FM models show that “observed choices in discounting experiments are consistent with roughly one‐half of the subjects using exponential discounting and one‐half using quasi‐hyperbolic discounting.” And using a Bayesian approach, Houser et al. show that different people use different decision rules—specifically, one of three different criteria—when solving dynamic decision problems (Houser et al., 2004).

Multiple choice criteria are also well established in the empirical‐game‐theory literature. Stahl and Wilson fit an FM model to data on play in several classes of 3 × 3 normal‐form games, and find that players fall into five different boundedly rational choice‐criteria classes (Stahl and Wilson, 1995). Guessing games—also known as Beauty‐Contest games—have been pivotal in showing not only that backward induction and dominance‐solvability break down, but also that game play can be characterized by membership in a boundedly rational, discrete (level‐*k*) depth‐of‐reasoning class (Nagel, 1995). FM models are the technique of choice for analyzing Beauty‐Contest data, revealing that virtually all “nontheorist” subjects^15^ (94%) fall into one of three boundedly rational depth‐of‐reasoning classes (level 0, 1, or 2) (Bosch‐Domènech et al., 2010; Stahl, 1996). FM models are being applied increasingly in empirical game theory—including to the analysis of, for example, trust‐game data, social‐preferences data, and common‐pool‐resource data—demonstrating the broad applicability of a multiple‐criteria approach. The theoretical relevance of level‐*k* reasoning to adversarial interactions such as phishing has been further demonstrated by Rothschild et al. (2012), however we know of no existing paper in this field that allows alternative choice criteria to coexist.

Outside decision theory and empirical game theory, the necessity of allowing for multiple choice criteria has also been recognized in the fields of transportation research and consumer research. Within a latent class (LC) model framework,^16^ Hess et al. (2012) study the question of whether “actual behavioral processes used in making a choice may in fact vary across respondents within a single data set.” Preference heterogeneity documented in conventional single‐choice‐criterion models^17^ may be a logical consequence of the single‐choice‐criterion restriction (i.e., misspecification). Hess et al. (2012) account for choice‐criterion heterogeneity in four different transport‐mode‐choice data sets by fitting LC models. These LC models distinguish between conventional random utility and the lexicographic choice criterion (data set 1), among choice criteria with different reference points (data set 2),^18^ between standard random utility and the elimination‐by‐aspects choice criterion (data set 3), and between standard random utility and the random‐regret choice criterion (data set 4). Finally, Swait and Adamowicz (2001) show that *consumers* also fall into different “decision strategy” LCs, and that increasing either the complexity of the choice task or the cumulative task burden induces switching toward simpler decision strategies. These results underscore an interpretation of the choice‐criterion probabilities that is only implicit in the above‐mentioned studies: that (a) decisionmakers should not be characterized solely in terms of their *modal* choice criterion, but in terms of their choice‐criterion mixtures, and that (b) the criterion that is operative for a particular choice task is obtained as a draw from the probability distribution over choice criteria, which in turn is conditional upon features of the context, the framing and presentation of the choice options, and the current psychological characteristics of the decisionmaker.

In light of these FM‐ and LC‐model findings, accommodation of multiple choice criteria emerges as a natural step toward improving the descriptive validity of theoretical models.

## 4 INCORPORATING INTUITIVE, EMOTIONAL, AND FALLIBLE DECISION MAKING

### 4.1 Coexisting‐choice‐criteria model

The econometric evidence reviewed in Section 3 warrants a generalization of decision theory to incorporate multiple coexisting choice criteria. An abstract formulation of such a theory naturally draws upon the formal specification of econometric LC models that capture choice‐criterion heterogeneity (Hess et al., 2012; Swait & Adamowicz, 2001).

Let C denote the set of coexisting choice criteria. The elements of this set are distinguished by the integer‐valued index *c*, where 1≤c≤C:=|C|.

We specialize the present formulation to the context of phishing‐security modeling by populating the set of choice criteria C with a view to capturing the essential features of human beings in the security setting, as reviewed in Section 2. Email recipients are capable of rational deliberation, but they are not overwhelmingly predisposed to it. They may instead form subjective beliefs and valuations as captured by behavioral decision theory, but they also frequently act in an intuitive or routinized fashion. Thus the empirical evidence reviewed in Section 2 suggests that human responses to phishing campaigns range across (at least) four identifiable choice criteria, which we summarize in Table 1.^19^

**TABLE 1:** Email recipients' coexisting choice criteria

c=1	Normative deliberation: characterized by the internal‐consistency axioms of completeness, transitivity, independence of irrelevant alternatives (iia), continuity, Bayesian updating, and time consistency (i.e., exponential discounting).
c=2	Behavioral: characterized by the weakening of iia, Bayesian updating, and time consistency (i.e., to hyperbolic discounting), as per the behavioral decision‐making literature.
c=3	Impulsively click through: characterized by dominance of visceral emotions, which suppress and displace deliberative reasoning; the remaining consistency axioms are abandoned.
c=4	Routinely click‐straight‐through: characterized by routinization and automaticity; again, the remaining consistency axioms are abandoned.

In general, the choice‐criterion selection probability will be conditional upon the decisionmaker's SoM, which in turn depends on an array of subject‐ and task‐specific variables. The joint effect of all such variables determines an individual's probability of adopting a given choice criterion *c* at a given point in time, which we denote by $\pi_{it}^{c}$. Note that we necessarily have $0\leq \pi_{\text{it}}^{c}\leq 1$ and $\sum_{c=1}^{C}\pi_{\text{it}}^{c}=1$ for all individuals *i* and time points *t*.

**FIGURE 1:**
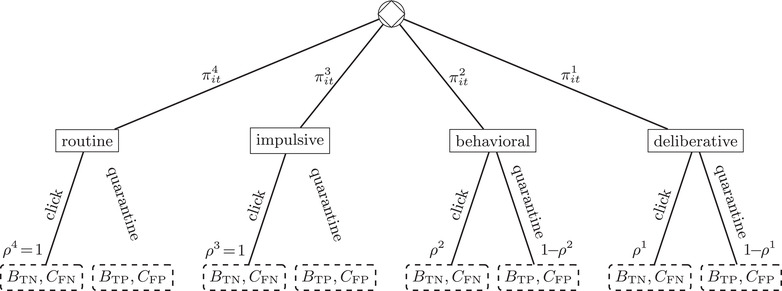
An agent's stochastic state‐of‐mind response to an email *Note*: *Ex ante* the agent is uncertain about an email's true nature. The payoff at each terminal node is therefore either a benefit due to correct classification (True Positive or True Negative), or a cost due to incorrect classification (FP or FN)

Figure 1 illustrates a single agent's stochastic SoM response to an arbitrary email. This begins with the diamond‐within‐a‐circle chance node, whereby the incoming email probabilistically triggers one of the four SoM choice criteria.^20^ The fact that the “Routine” (c=4) and “Impulsive” (c=3) choice criteria override the possibility of sufficient deliberation to result in a “quarantine” choice with probability ρ=0 is indicated by the absence of these respective edges. This is a simplification. For instance, fat‐finger mistakes can lead to an ε>0 probability of quarantine. Introducing this explicitly into Figure 1 and Table 2 would have the effect of complicating subsequent mathematical expressions, but it would not change the essence of the results of the analysis carried out in Section 4. The mechanisms by which strong visceral emotions suppress and displace deliberative reasoning are discussed above in Section 2. In summary, strong visceral emotions (i) narrow the range of both attention and thinking to the visceral factor itself and its remedy, and (ii) induce a sense of being “out of control” in which “action is driven by instinct and gut feelings, and careful analysis is abandoned” (Langenderfer & Shimp, 2001; Loewenstein, 1996).

**TABLE 2:** Choice‐criterion targeting characteristics

Choice criterion	Effort	Click‐through prob.	Selection prob.^a^	
*c*	$e(\underset{\alpha }{\mathrm{argmax}}\left\{\pi^{c}\right\})$	$\rho^{c}$	Prior	Posterior
c=1: Deliberative	Low	Negligible	High	High
c=2: Behavioral	Low	Low	Med	Med
c=3: Impulsive	Low	1	Low	Low
c=4: Routine	High	1	Low	High

^a^That is, $\max_{\alpha }\left\{\pi^{c}\right\}$.

The email recipient's incomplete information—over whether the email is benign or malicious—is reflected in the broken‐line information sets surrounding terminal‐node payoffs.

The email recipient is one of many agents who interact in a strategic phishing game. We analyze an attacker's optimal response to Figure 1 in Section 4.3, and we discuss the model's implications for organizational security policy in Section 4.4. Before doing so, we complete the model by expanding the $\pi_{it}^{c}$ expression for an agent *i* at time *t*. In general, $\pi_{it}^{c}$ is operationalized through a probability distribution that may be conditional upon: the characteristics of the decisionmaker $X_{it}$, the situational context $Z_{it}$, and the attributes of the present choice task $\alpha_{t}$.

(1a) $$\begin{array}{c} \begin{array}{c} \pi_{\mathrm{it}}^{c}=\pi^{c}\left(X_{\mathrm{it}},Z_{\mathrm{it}},\alpha_{t}\right),\;0\leq \pi_{\mathrm{it}}^{c}\leq 1,\;\sum_{c=1}^{C}\pi_{\mathrm{it}}^{c}=1, \end{array} \end{array}$$

(1b) $$\begin{array}{c} X_{it}=f\left(\Gamma_{i},{\left\{Z_{i}\right\}}_{<t},{\left\{\alpha \right\}}_{<t},{\left\{D_{i}\right\}}_{<t}\right). \end{array}$$

The current characteristics $X_{it}$ of agent *i* are jointly determined by their stable psychological traits $\Gamma_{i}$, and by the history of: decision contexts ${\left\{Z_{i}\right\}}_{<t}$, decision‐attributes ${\left\{\alpha \right\}}_{<t}$, and decision‐outcomes ${\left\{D_{i}\right\}}_{<t}$ that constitutes their current set of experiences.

In order to develop a tractable expression for $\pi_{it}^{c}$ we generalize the notion of match quality introduced in the SDT literature (Kaivanto, 2014) and we specialize the vectors appearing in (1a) to the phishing‐email application. For this application, the context $Z_{it}$ is that in which the agent receives his emails. An agent whose context $Z_{it}$ and recent context history ${\left\{Z_{i}\right\}}_{<t}$ leaves him stressed, distracted, or hungry, will be less likely to respond deliberatively. The implications of this observation for personal practice and organizational security policy are clear,^21^ and so we suppress $Z_{it}$ hereafter to focus on the strategic interaction between attackers and recipients. For simplicity, we also suppress time subscripts hereafter to focus on the short‐run implications of the model.

Let us consider a phishing email with attribute vector ***α*** constructed within a finite attribute space $A={\left[0,1\right]}^{A}$, where *A* is the total number of possible cues and thus also the number of components in the attribute vector ***α***. The attacker chooses which cues to emphasize in order to influence the recipient's SoM. For example, the attacker may target a routine choice criterion by impersonating a client, or she may target an impulsive choice criterion by means of an urgent and attractive “special offer.” This determination of email “content” is the attacker's primary decision variable.

The attacker is nevertheless constrained, in that increasing the emphasis placed on any one cue necessarily diminishes the emphasis on the others. We model this constraint by requiring ||α||≤1.

The salient characteristics of the recipient are his idiosyncratic susceptibility to each type of cue $S_{i}$, and his baseline propensity $\chi_{i}^{c}$ to apply each choice criterion *c*.^22^ $S_{i}$ is an C×A dimensional matrix, each row of which $s_{i}^{c}$ specifies the effectiveness of each possible cue type in invoking the choice criterion *c*. The agent's characteristics $X_{i}$ are therefore a matrix in ${\left[0,1\right]}^{A\times C}\times {\left(R^{+}\right)}^{C}$, each row of which is a pair $\left\{s_{i}^{c},\chi_{i}^{c}\right\}$ that will determine the match quality between the attacker's choice of email cues ***α***, and the susceptibilities of the receiving agent *i*.

We may now extend the approach of Kaivanto (2014) by defining the choice‐criterion‐specific match‐quality function $m^{c}\;:\;{\left[0,1\right]}^{A}\times {\left[0,1\right]}^{A}\times R^{+}\to R^{+}$, such that

(2) $$m_{i}^{c}\left(\alpha \right)=m^{c}\left(\alpha ,s_{i}^{c},\chi_{i}^{c}\right)\;\forall c\in C.$$

For illustrative purposes, the simplest nondegenerate functional form for $m^{c}$ would be the separable linear specification

(3) $$m_{i}^{c}\left(\alpha \right)=\chi_{i}^{c}+s_{i}^{c}\cdot \alpha ,$$

where · denotes the vector dot product.

Agent *i*'s choice‐criterion‐selection probabilities for a given email with cue bundle ***α*** may then be defined in terms of the match‐quality functions as follows:

(4) $$\pi_{i}^{c}\left(\alpha \right)=\frac{m_{i}^{c}\left(\alpha \right)}{\sum_{c\in C}m_{i}^{c}\left(\alpha \right)}\;\forall c\in C.$$

As noted above, Equations (1b) through (4) build on the conceptual apparatus introduced in the SDT‐based phishing susceptibility literature (Kaivanto, 2014). There are close parallels between the concepts employed here—histories of decision contexts ${\left\{Z_{i}\right\}}_{<t}$, decision‐attributes ${\left\{\alpha \right\}}_{<t}$, decision‐outcomes ${\left\{D_{i}\right\}}_{<t}$, as well as match quality $m_{i}^{c}\left(\alpha \right)$—and those employed in IBLT (Cranford et al., 2019; Gonzalez et al., 2003). Hence, the machine learning and NLP techniques developed in IBLT can in principle be used in computation implementation of (4).^23^

### 4.2 Contrast with normatively rational deliberative special case

Under a normative decision‐theoretic model of email‐recipient decision making, it is difficult to explain the existence of phishing as an empirical phenomenon. Normatively rational decision making is a special case of the coexisting‐choice‐criteria model in which $\pi^{1}=1$ and $\pi^{2}=\pi^{3}=\pi^{4}=0$. If all email recipients were characterized by choice‐criterion #1 alone, then the success of an email phishing campaign would be determined entirely by factors largely outside the attacker's control: the benefit from correctly opening a nonmalicious email ($B_{\text{TN}}$), the cost of erroneously quarantining nonmalicious email ($C_{\text{FP}}$), the cost of erroneously opening a malicious email ($C_{\text{FN}}$), and the benefit of correctly quarantining a malicious email ($B_{\text{TP}}$). Instead, variation in phishing campaigns' success rate is driven by factors that do not directly affect $B_{\text{TN}},C_{\text{FP}},C_{\text{FN}}$, and $B_{\text{TP}}$ (Hadnagy, 2011; Mitnick & Simon, 2002; Oliveira et al., 2017; Rusch, 1999).

It is straightforward to explain the existence of phishing and its empirical characteristics under a coexisting‐choice‐criteria model of email‐recipient behavior in which $\pi^{1}<1$ and $\pi^{2},\pi^{3},\pi^{4}>0$. For instance, choice‐criterion #4 (routine, automaticity) is triggered by a phishing email that masquerades as being part of the normal work flow by exploiting rich contextual information about the employee, the organizational structure (e.g., boss' and colleagues' names, responsibilities, and working practices), and current organizational events and processes. Here, the email recipient simply does not engage in a deliberative process to evaluate whether the email should be opened or not.^24^

In contrast, phishing ploys designed to trigger choice criterion #3 (impulsively click through) employ what Robert Cialdini calls the *psychological principles of influence* (see Section 2) (Cialdini, 2007; Hadnagy, 2011; Mitnick & Simon, 2002; Oliveira et al., 2017; Rusch, 1999). Importantly, there is variation between individuals in their susceptibility to particular levers of psychological influence (Oliveira et al., 2017; Vishwanath et al., 2011; Williams et al., 2017). For instance scarcity^25^ and authority^26^ have been found to be more effective for young users, while reciprocation^27^ and liking/affinity^28^ have been found to be more effective for older users (Oliveira et al., 2017). These observations motivate the agent‐specific subscript *i* in $\pi_{i}^{c}$ and $m_{i}^{c}$, and they are important in establishing the constrained‐optimal APT attack pattern in the following subsection.

None of the aforementioned psychological levers would be effective if email users were solely c≡1 normatively rational deliberators. Similarly, the well‐documented effects of commitment,^29^ perceptual contrast,^30^ and social proof^31^ (see Hadnagy, 2011; Mitnick & Simon, 2002; Oliveira et al., 2017; Rusch, 1999) are naturally explained by the existence of coexisting choice criteria.

### 4.3 Stepping‐stone penetration

Forensic investigations of APT attacks have found that the initial breach point is typically several steps removed from the ultimate information‐resource target(s) (Bhadane & Mane, 2019; Quintero‐Bonilla & del Rey, 2020). Deliberation‐based models of normatively rational decision making offer no particular insight into APT attacks' use of social engineering to gain an initial foothold, followed by lateral movement within the organization.^32^ In contrast, as we show below, the coexisting‐choice‐criteria model encodes differentiation with which the stepping‐stone penetration pattern may be recovered as a constrained‐optimal attack vector.

Let us consider an attacker who wishes to achieve a click‐through from one of a minority subset of *m* target individuals within an organization consisting of *n* members. The target individuals may be those who can authorize expenditure, or those with particular (e.g., database) access rights. The attacker's strategy at any given point in time consists of a choice of cue‐bundle $\alpha_{k}$, taken to solve the program

(5) $$\max_{\alpha_{k}}\sum_{i=1}^{m}\sum_{c=1}^{C}\pi_{i}^{c}\left(\alpha_{k}\right)\cdot \rho_{i}^{c}\cdot V-e\left(\alpha_{k}\right)\;\text{s.t.}\;||\alpha_{k}\left|\right|\leq 1\;,$$

where $\pi_{i}^{c}\left(\alpha_{k}\right)$ is the probability with which an individual will adopt choice criterion *c* given the cues present in phishing email $\alpha_{k}$, where $\rho^{c}$ is the probability of click‐through given choice criterion *c*, where *V* is the expected value of a successful attack, and where $e(\alpha_{k})$ is the cost of the effort expended in the production and distribution of email $\alpha_{k}$. This formulation accords with the near‐zero marginal cost of including additional recipients to any existing email (Anderson, 2008; Shapiro & Varian, 1998).

The attacker may send one, or more, emails $\alpha_{k}$. Each email may be designed to induce one particular SoM *c*, or could in principle adopt a mixed strategy. However, since (by construction and by necessity) $\sum_{c\in C}\pi_{i}^{c}=1$, any mixture of asymmetrically effective pure strategies must be strictly less effective than at least one pure strategy. We therefore proceed by characterizing the available pure strategies on the basis of the phishing literature (Hadnagy, 2011; Mitnick & Simon, 2002; Rusch, 1999), before eliminating strictly dominated strategies.

The quantities summarized in Table 2 determine the costs and expected benefits to the attackers of targeting choice criterion *c* through their choice of ***α***. There are two values of the selection probability $\max_{\alpha }\left\{\pi^{c}\right\}$ for each choice criterion *c*: the prior likelihood of invoking that criterion, without insider information, and the posterior likelihood once access to such insider information is obtained. Insider information does not affect the attacker's ability to invoke choice criteria c∈{1,2,3}, but it does greatly aid the attacker's ability to “spoof” (i.e., simulate) a routine email from a trusted colleague, and hence it substantially increases the posterior selection probability for c=4. The mechanism by which attackers may gain such insider information is the successful phishing of a nontarget member of the organization.

The most immediate implication of Equation (5) and Table 2 is that the Deliberative strategy is strictly dominated by the Behavioral strategy, due to the negligible click‐through probability of the former. We next observe that the Behavioral strategy is, in turn, strictly dominated by the Impulsive strategy whenever

(6) $$\rho^{2}<\frac{\max_{\alpha }\left\{\pi^{3}\right\}}{\max_{\alpha }\left\{\pi^{2}\right\}}.$$

That is, whenever the expected click‐through probability under a Behavioral choice criterion is less than the relative ease of invoking the Behavioral state compared to invoking the Impulsive state. Table 2 suggests that this criterion is typically satisfied.

Next we consider the case of an attacker who has no insider information. In this case, it is trivial to see that an email which aims to invoke the Impulsive choice criterion strictly dominates an email which aims to invoke the Routine choice criterion, due to the lower effort cost of the former. The respective probabilities of successfully gaining a click‐through from a target individual are then:

(7) $$\begin{array}{ccc} & & \text{Prob.}\left(\begin{array}{c} \text{nontarget}\\ \text{click-through} \end{array}\right)=1-{\left(1-\max_{\alpha }\left\{\pi^{3}\right\}\right)}^{n-m}\\ & & \;>\;1-{\left(1-\max_{\alpha }\left\{\pi^{3}\right\}\right)}^{m}=\text{Prob.}\left(\begin{array}{c} \text{target}\\ \text{click-through} \end{array}\right),\; \end{array}$$

which demonstrates that there is a greater likelihood of the attacker gaining a click‐through from a nontarget individual than from a target individual in any attack without insider information. Note that this conclusion would be further strengthened if we were to assume that target individuals were less susceptible to phishing attacks than the average individual.

The attacker's first attempt therefore has three possible outcomes: (i) they may have successfully achieved their objective, (ii) they may have gained insider information by achieving a nontarget click‐through, or (iii) they may have achieved nothing. In the first case, the attacker move on to acquire and exfiltrate the information. In the third case, the situation is unchanged, and so the phishing campaign is continued with further broadcast of phishing email(s) containing (possibly modified) Impulsive cues. But in the second case insider information is obtained, whereby the posterior click‐through likelihoods of Table 2 become operative. In this case, it is evident from Table 2 that an email which aims to invoke the Routine choice criterion is likely to dominate an email which aims to invoke the Impulsive criterion, specifically whenever

(8) $$\frac{e\left({\text{argmax}}_{\alpha }\left\{\pi^{4}\right\}\right)}{e\left({\text{argmax}}_{\alpha }\left\{\pi^{3}\right\}\right)}<\frac{\max_{\alpha }\left\{\pi^{4}\right\}}{\max_{\alpha }\left\{\pi^{3}\right\}}.$$

Thus the attacker's optimal approach is likely to lead to a “stepping‐stone” attack, wherein a nontarget individual is first compromised by invoking an impulsive choice criterion, so that a target individual can then be compromised by using insider information to invoke a Routine choice criterion. Sufficient conditions for this to be the most likely outcome are those of Table 2 and inequalities (6) and (8).

### 4.4 Implications for organizational security policy

The model we present has important implications for organizational security policy. Let us first consider the cultural and procedural aspects of organizational security, before turning to specific implications for email security training and evaluation.

In Section 4.1, we noted the potential importance of the situational context $Z_{it}$ in which an email is received. For example, it is well‐known that an individual who is under intense time‐pressure is less likely, if not simply unable, to engage in deliberative decision making (Hwang, 1994; Maule & Edland, 1997; Steigenberger et al., 2017). The present model makes plain the security‐vulnerability dangers of highly routinized email‐processing practices, even if these would otherwise be efficient. Relatedly, it is vital that organizational culture supports the precautionary verification of suspicious messages, since any criticism of such verification practices is likely to increase the risk of behavioral click‐throughs in future. These observations suggest that ISOs should actively engage with wider aspects of organizational culture and practices.

The model also yields specific procedural implications for email security training. It is clear that the direct effect of a training course in which participants consciously classify emails as either genuine or malicious would be to reduce ρ^1^ (see Figure 1), however for most individuals ρ^1^ is already relatively low (see Table 2): given that an individual implements a deliberative choice criterion they are relatively unlikely to fall prey to a phishing attack. Section 4.3 demonstrated that a strategic attacker would instead seek to exploit the much greater vulnerabilities of ρ^3^ and ρ^4^, and so training that focuses on reducing ρ^1^ is likely to have limited effectiveness.

The challenge for ISOs is that the vulnerabilities ρ^3^ and ρ^4^ are essentially fixed at 1.^33^ Once an Impulsive or Routine SoM takes over, click‐through is a foregone conclusion. Training should therefore focus on reducing individuals' criterion‐selection probabilities π^3^ and π^4^. There is evidence that an individual's propensity to act deliberatively can be raised through external interventions (Frederick, 2005), and the coexisting‐choice‐criteria framework suggests that this could best be achieved by helping employees to understand:

(i) their inherent vulnerability to phishing when making choices either Routinely or Impulsively, and
(ii) the psychological ploys by which attackers may induce Impulsive or Routine SoM.

Analogous implications exist for procedures which aim to test organizational security by means of simulated phishing emails. Where such a test is appended to a training module, it tests (at best) some combination of ρ^1^ and ρ^2^, because trainees will be aware that they are attempting to identify phishing emails. Furthermore, the literature on incentives suggests that where such a test is incentivized with some required pass‐rate, it is likely to be less informative as to the true vulnerability level because it is more likely to generate a pure measure of ρ^1^. Tests of security should therefore be blinded, for example, by an unannounced simulation of an email attack. Moreover, such tests should be varied and repeated, since any single email ***α*** can only contain one specific cue bundle, and so can only test an individual's susceptibility $\pi^{c}\left(\alpha \right)$ to that particular cue bundle.

## 5 TRAINING EXPERIMENT

What forms of training interventions are implied by the coexisting‐choice‐criteria model, and how effective are they?

### 5.1 Design and procedures

Intervention design proceeds within bounds set by time and resource constraints. If such constraints were absent, an intervention could aim to enhance the quarantine probability under each of the four choice criteria. Yet the quarantine probability under the deliberative choice criterion is high even in the absence of intervention, whereas the quarantine probabilities under impulsive and routine choice criteria are zero (see Table 2). The quarantine probability under the behavioral choice criterion is closer to that under deliberation than under either impulsive or routine choice criteria. Our model therefore implies that an individual's overall phishing‐email detection performance may be improved most effectively by interventions that alter the SoM distribution rather than by industry‐standard interventions that aim to raise the quarantine probability while in the deliberative SoM. The greatest incremental benefit is obtained from interventions that decrease $\pi_{i}^{4}$ or $\pi_{i}^{3}$ (or both) while increasing $\pi_{i}^{1}$—that is, by decreasing the probability of routine or impulsive choice criteria (or both) while increasing the probability of the deliberative choice criterion.

We therefore design a pair interventions, each of which aims to shift probability mass within the SoM distribution: one designed to shift probability mass from routine to deliberative, the other designed to shift probability mass from impulsive to deliberative.

In implementing these training packages, special care is devoted to grabbing the viewer's attention and to showing them that they too are susceptible to impulsive (in *TII*) and routine (in *TRI*) response modes. Then the viewer is introduced to simple strategies they can adopt to ensure a more deliberative approach to gauging the risk posed by an email and to dealing with it safely.

*Baseline Treatment* (*TB*). An established 10‐min multimedia interactive training module developed and refined by the information security and information systems training teams of Lancaster University. It provides detailed yet accessible training on the skills needed to identify phishing emails and websites. As an industry‐standard training module, it is premised on the assumption that individuals draw upon these skills when receiving incoming messages—that is, it assumes that individuals process emails while in a deliberative SoM.

*Routine‐Interrupt Treatment* (*TRI*). A 7‐min multimedia interactive training module with periodic understanding‐verification questions. This training video is designed to cause the student to slow down, think consciously, and preempt “routine” and “automatic” processing of email information. The transcript of this training video is presented in Appendix A.

*Impulse‐Interrupt Treatment* (*TII*). An 8‐min multimedia interactive training module with periodic understanding‐verification questions. This training video is designed to (a) alert the student to those features of an email that aim to elicit visceral emotions and thereby trigger an impulsive response, and to (b) provide strategies for processing emails to reduce the likelihood of impulsive clicks. The transcript of this training video is presented in Appendix B.

*TRI* and *TII* share some common elements. Slides 1 and 2 of *TRI* and *TII* are identical. On slide 3, the two treatments differ only in the content of the very last sentence. The last sentence on slide 3 sets up the approach that is elaborated in the remaining slides. In the *TRI* training package, the sentence posits that susceptibility to phishing attacks “could also be because experience and efficiency in dealing with electronic messages is, itself, our greatest weakness.” The subsequent four slides elaborate and illustrate the phishing vulnerability associated with automatic email processing routines, and the value of selectively slowing those processes down sufficiently to perform rudimentary authenticity checks (e.g., hovering over links). In the *TII* training package, the sentence posits that susceptibility to phishing attacks “could also be because our weaknesses do not lie in a lack of ability to recognise phishing scams, but rather in our human psychology.” The subsequent four slides elaborate and illustrate the phishing vulnerability associated with impulsive, visceral emotion driven email processing, and the value of slowing and interrupting this response mode.

*Subjects*. Subjects were recruited from Lancaster University's student population through an email inviting students to take the University's antiphishing training. Participation was voluntary. The *TB* training package was the institution's existing status quo training package. Alongside this, we introduced two alternative training packages: *TRI* and *TII*. Students who accepted the invitation were randomly allocated to *TB*, *TRI*, or *TII*. In total, 332 student subjects gave informed consent and completed their assigned training module along with the posttraining test: 108 under the *TB* control condition, 110 under the *TRI* routine‐interrupt treatment, and 114 under the *TII* impulse‐interrupt treatment. Thus the treatment groups are balanced, with the largest and smallest groups deviating no more than 3% from the mean.

*Procedures*. The training package and follow‐up test were administered to participants via the university's Virtual Learning Environment (Moodle). The follow‐up test consisted of a landing & introduction page, a consent page, six test‐email pages (see Table 3), and a final outro page. Each test‐email page contains not only the html‐formatted test email complete with embedded links, but also a further section asking the participant to rate “How likely would you be to fall for an attack like this in real life?” with radio buttons for 95%, 75%, 50%, 25%, and 5%.

**TABLE 3:** Test emails

Description	Weakness targeted	Authenticity
justWink e‐card	Impulsive response	Genuine
Facebook notification	Routine response	Genuine
Library renewal reminder	Routine response	Fake
Student union discount card offer	Impulsive response	Fake
Law enforcement fraud alert	Impulsive response	Fake
Antiphishing project collaboration invitation	Routine response	Fake

### 5.2 Results

Three summary measures are calculated for each participant: *score* is the global performance summary measure: the proportion of correctly identified emails; *true pos* is the proportion of phishing emails correctly identified as such; *true neg* is the proportion of genuine emails correctly identified as such. In addition, *confidence* is the participant's average self‐assessed probability of correctly identifying phishing emails in real life.

**FIGURE 2:**
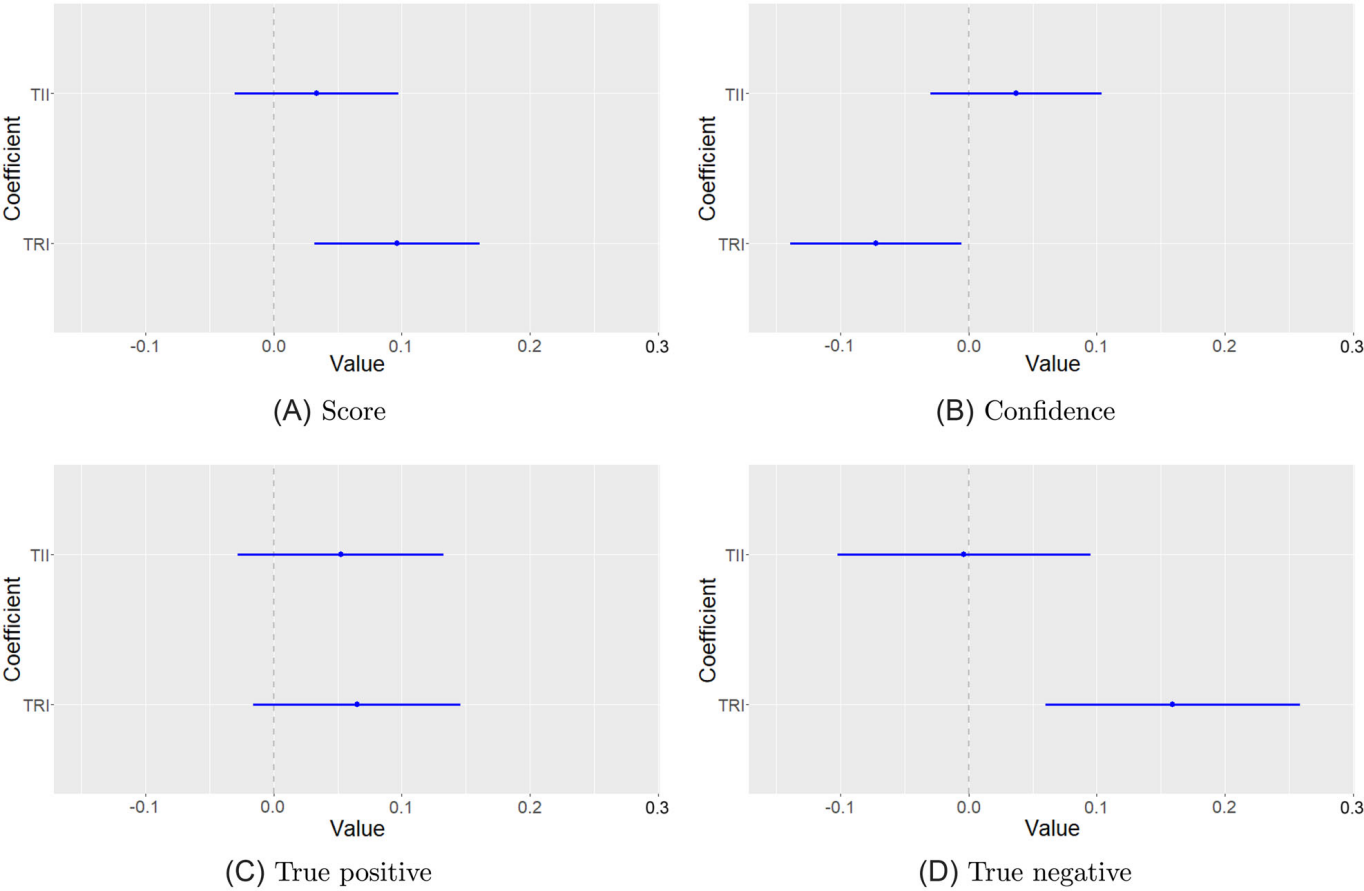
Plot of *TRI* and *TII* parameter estimates with confidence intervals, for each of the four linear probability models

We estimate the mean marginal effect of each treatment condition on these outcome measures using a linear probability model (LPM). Whereas LPM is vulnerable to functional‐form misspecification in the case of continuous predictor variables, here that concern does not apply because treatment status is a discrete indicator variable. The advantage of using a LPM to analyze randomized controlled experiment data is that the parameter estimates are straightforwardly interpreted as the mean marginal effect on the outcome.^34^ As the sample is balanced across treatment groups (within 3% of the mean) heteroskedasticity is not a problem *a priori*. Reestimating the LPMs with robust standard errors has no effect on the empirical results nor on the associated levels of statistical significance. In Table 4, we report the LPM estimates along with their robust standard errors. Figure 2 presents the coefficient information visually.

**TABLE 4:** Linear probability model parameters and standard errors

	Outcome measures			
	*score*	*true pos*	*true neg*	*confidence*
*TB*				
*TRI*	0.0965	0.0650	0.1593*	−0.0724*
	(0.0323)	(0.0411)	(0.0491)	(0.0352)
*TII*	0.0336	0.0523	−0.0037	0.0371
	(0.0312)	(0.0405)	(0.0493)	(0.0318)
*constant*	0.5278*	0.5486*	0.4861*	0.6433*
	(0.0226)	(0.0299)	(0.0345)	(0.0239)
*n*	332	332	332	328
*R* ^2^	0.0273	0.0087	0.0413	0.0334

*Note*: *, and * denote statistical significance at the 5%, 1%, and 0.1% levels, respectively.

**FIGURE 3:**
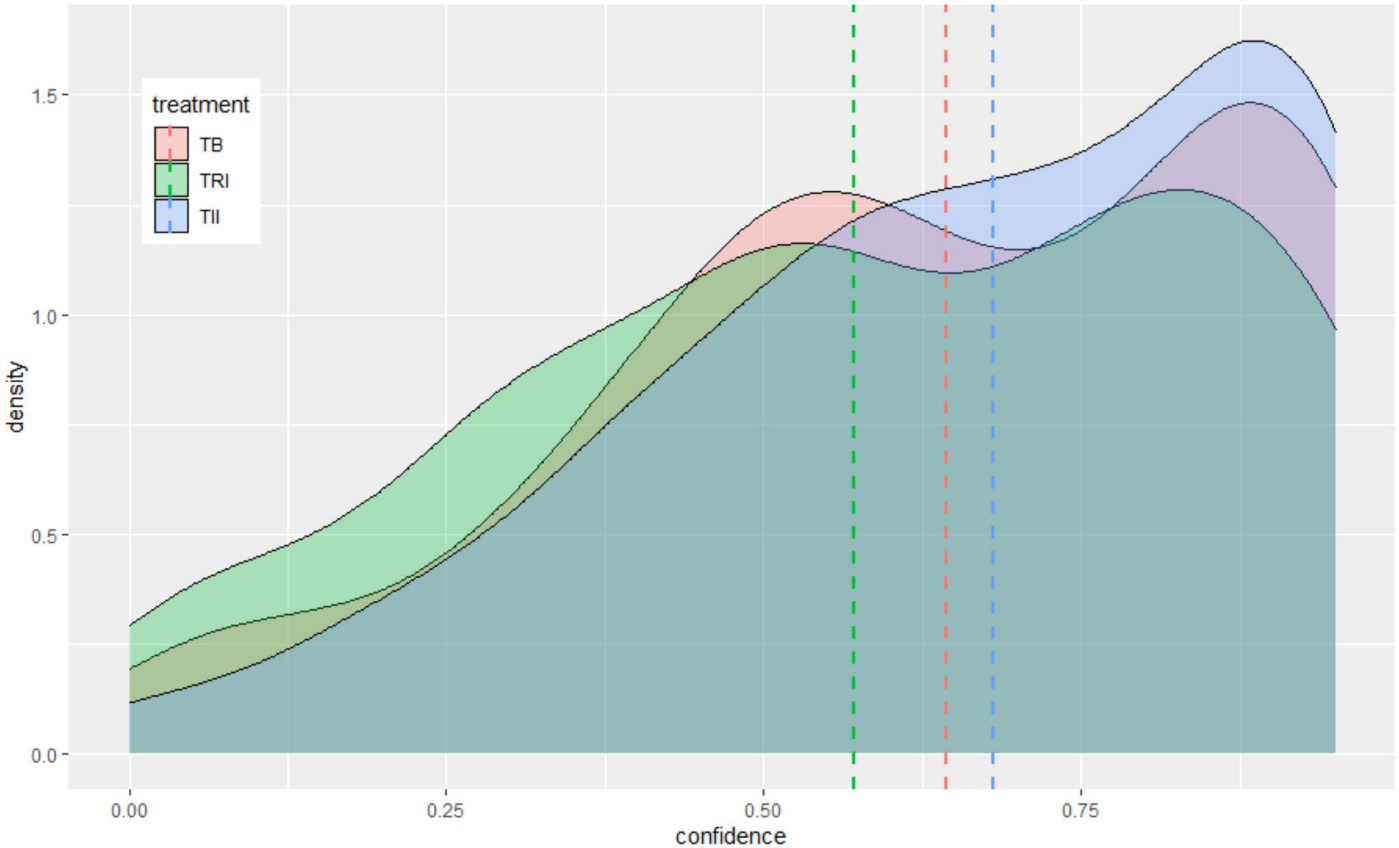
Distribution of confidence according to treatment status, with means indicated by dashed lines

Relative to the benchmark industry standard training package (*TB*), the routine email processing interrupt treatment (*TRI*) has a statistically significant effect on overall email classification performance (*score*), the proportion of genuine emails correctly classified as such (*true neg*), as well as self‐assessed probability of correctly identifying phishing emails in real life (*confidence*). Those participants assigned to the *TRI* training package correctly classified nearly 10% more emails than those assigned to the benchmark training package. The *true neg* regression shows that this improved performance is associated with *TRI* participants correctly identifying genuine emails at a much higher rate (16%) than the group receiving the benchmark training package. Whereas the impulsive response interrupt treatment (*TII*) has no statistically significant effect on self‐assessed confidence, *TRI* has a negative effect on self‐assessed confidence (−7%), which is significant at the α=0.05 level. Although a separate study would be required to clarify the position and role of confidence in the causal chain between *TRI* and improved phishing email classification, one interpretation of the results in Table 4 and the distributions in Figure 3 is that individuals who take packages *TB* or *TII* are overconfident in their ability to avoid falling victim to phishing attacks in real life, whereas the *TRI* treatment disabuses some of that overconfidence. Figure 3 is diagnostic of the refinements needed for *TII* to become effective.

These results suggest that training programs informed by the coexisting‐choice‐criteria model can provide a statistically significant improvement in personal and organizational security risk. This experiment shows *how* the model may be used to guide the design of antiphishing training: by encouraging individuals to recognize their routine and/or impulsive email‐processing responses and to adopt approaches that supplant these responses with conscious deliberation. The two antiphishing training packages implemented here were not equally successful, however, with the routine‐interrupt (*TRI*) package outperforming the impulsive‐interrupt (*TII*) package as well as the industry‐standard (*TB*) baseline package. This suggests that future versions of the standard antiphishing training package can benefit most from incorporating elements embodied in the (*TRI*) package, including deterrence of (over)confidence.^35^ Experiments have shown that deliberative reasoning can be “activated by metacognitive experiences of difficulty or disfluency during the process of reasoning” (Alter et al., 2007), that is, by experiences that affect confidence negatively.^36^

*TII* subjects come out of their training just as confident (in distribution) as *TB* subjects, and their overall classification performance, true‐positive performance, and true‐negative performance is statistically indistinguishable from the *TB* status quo. This classification performance itself could be the result of *either* or *both* (i) fundamental difficulty of interrupting the impulsive SoM, or (ii) failure of *TII*'s design to stimulate metacognitive processes. The confidence data in Figure 3 is consistent with (ii) and a failure of *TII* to interrupt perceived fluency and absence of difficulty. However, the confidence data does not rule out (i) either.

*TRI* on the other hand is a limited success, in that it increased the true‐negative classification rate relative to *TB* by 15%. But it did not have a statistically significant effect on the true‐positive classification rate relative to *TB*. *TRI* appears to be successful in stimulating metacognitive processes, and relatedly, in reducing (over)confidence.

An anonymous reviewer has suggested an alternative interpretation of the experimental results. If it is assumed that all participants are in the deliberative SoM throughout the phishing email identification task, then the study only shows that the TRI condition increases the probability of clicking links in safe emails (i.e., the true negative probability increases). The reduction in Confidence among those receiving TRI could then be attributed to an increase in the number and unfamiliarity of features upon which they are making their judgments, and not to an increase in metacognitive activity. In order to distinguish cleanly between the two interpretations, the phishing email identification task would need to be carried out as a field experiment, where the mock phishing emails are sent to participants' email accounts and would therefore be processed as part of their normal email correspondence without any prior priming for deliberation save for the original TRI treatment. Further research of this nature is needed to fully reveal the effectiveness of training interventions inspired by the coexisting‐choice‐criteria model.

Can training, enhanced with the insights of the coexisting‐choice‐criteria model, ultimately solve an organization's phishing‐susceptibility problem? Not fully. But the coexisting‐choice‐criteria model suggests complementary measures that can help organizations reach that goal.

Although visceral emotions can be powerful, they are short‐lived. Hence, measures that allow or require a “cooling‐off period” can help mitigate phishing ploys targeting the impulsively click‐through choice criterion.

Another measure that can reduce susceptibility to phishing is to replace the nonstructured, informally developed email‐management routines with one that is structured and explicitly designed—perhaps using procedural checklists, as widely employed in safety‐ and security‐critical roles. Automation may speed and aid use of such checklists. Such a procedural checklist effectively becomes another “choice criterion” c=5 in C.

Finally, security culture can be explicitly integrated into organizational culture. This can involve organizationwide cultural norms requiring, for example, cooling‐off periods and procedural checklists. Effectively establishing the procedural checklist as a cultural norm raises the probability of this choice criterion being selected, $\pi_{it}^{5}$, to a very high level. Norms of organizational culture can also involve the adoption of practices that strongly deprecate file attachments or links in internal email communications. Files and links can be shared through other secure means: for instance, via platforms such as MS Teams, access to which may be controlled by two‐factor authentication.

## 6 CONCLUSION

As the basis for understanding and modeling the behavior of phishing targets, normative deliberative rationality proves inadequate. This article introduces a coexisting‐choice‐criteria model of decision making that generalizes both normative and “dual process” theories of decision making. We show that this model offers a tractable working framework within which to develop an understanding of phishing‐email response behavior. This offers an improvement over existing SDT‐based models of phishing‐response behavior (Canfield & Fischhoff, 2017; Kaivanto, 2014), insofar as it avoids the commingling of peripheral‐route‐persuasion pathways.

We also show that the proposed framework may be usefully deployed in modeling the choices and tradeoffs confronted by APT attackers, who must make decisions about the nature, composition, and roll‐out of phishing campaigns. We illustrate this by tackling a problem that has confounded conventional normative‐rationalty‐based modeling approaches: Why do so many APT attacks follow a “stepping‐stone” penetration pattern? Under the coexisting‐choice‐criteria model, the attacker faces a tradeoff between (i) designing an email that is highly targeted, invokes the “Routine” choice criterion, but requires detailed inside information, and (ii) designing an email that cannot be targeted as effectively, invokes the “Impulsive” choice criterion, and requires only public information. However, success with (ii) provides the attacker with access to the inside information with which to implement (i). Thus, the stepping‐stone attack vector arises out of the attacker's tradeoffs precisely when confronting email users whose behavior is captured by the coexisting‐choice‐criteria model.

We further demonstrate that the model provides new insights with practical relevance for ISOs. We derive specific recommendations for information training and testing as well as for organizational procedures, practices, and policies. In particular, the model highlights the importance of considering the composite between the probability of being induced into SoM *c* and the probability of then clicking through *given* this SoM. Hence, training must address the different SoM selection probabilities $\pi^{c}$ as well as the associated conditional click‐through probabilities $\rho^{c}$. Analogously, security‐risk assessment processes will only be effective if they test the full range of vulnerabilities associated with the set of distinct SoM choice criteria. In light of the coexisting‐choice‐criteria model, the single‐test‐email approach should be deprecated.

Finally, the coexisting‐choice‐criteria model highlights organizations' vulnerability to spear‐phishing attacks that invoke automatic email processing routines. Working practices in most commercial, voluntary, and public‐sector organizations presume that links and email attachments are benign when sent from within the organization or by customers, suppliers, or partner organizations. This is a major vulnerability that is as much a reflection of organizational culture as it is a reflection of explicit security protocols (or absence thereof). ISOs could—and perhaps should—be afforded a broader role in shaping organizational culture. This could extend to establishing organization‐wide norms for the steps to be taken in vetting email as “low‐risk,” or in deprecating the sending of files as email attachments, sharing them instead via platforms such as MS Teams which can be protected with two‐factor authentication.

## Footnotes

1 In a phishing attack, a network user receives an email containing either an attachment or a website link, which if opened, prompts the user to enter personal information (e.g., passwords) or infects the user's computer with malware that records such information surreptitiously. For SDT‐based models of phishing vulnerability, see Kaivanto (2014) and Canfield and Fischhoff (2017).

2 The standardized distance between the means of the sampling distributions, respectfully under the null and alternative hypotheses.

3 The theory we present here is a specialization of Iain Embrey's “States of Mind and States of Nature” formulation (Embrey, 2017).

4
$C_{\text{Here}}⊃C_{\text{DP}}⊃C_{\text{EU}},\;C_{\text{PT}}$.

5 For example, commercial, administrative, professional service, and higher‐education organizations.

6 For instance, the production of research articles involves multiple exchanges of emails among coauthors themselves, between the coauthors and the journal's editorial team, and then between the coauthors and the publisher's production team. These emails contain file attachments, and sometimes URLs as well. In these exchanges, there is no security procedure in place to authenticate emails, their attachments, or embedded URLs.

7 In this penetration pattern, the attacker moves laterally within the organization, taking control of accounts and computers with the purpose of gaining knowledge of the organization's internal procedures and getting closer to the ultimate aims of the APT attack: access to or control over specific systems and/or information (Bhadane & Mane, 2019). In‐between the original breach point and the ultimate target(s) of the attack are stepping‐stone accounts and computers.

8 Sapience denotes wisdom and the capacity for sound judgment, particularly in complex or difficult circumstances.

9 For instance, the nonoperational computer systems of Wolf Creek Nuclear Operating Corporation in Kansas were penetrated (Perlroth, 2017).

10 as well as “hard‐sell” and “high‐pressure” marketing more generally.

11 A former swindler relates the principle: “It is imperative that you work as quickly as possible. Never give a hot mooch time to cool off. You want to close him while he is still slobbering with greed” (Easley, 1994).

12 We thank an anonymous reviewer for bringing IBLT to our attention, and for pointing out its potential for operationalizing coexisting‐choice‐criteria models.

13 Estimating the FM model yields a probability distribution over the set of choice criteria for each decisionmaker. It is common practice for researchers to categorize and label decisionmakers by their modal choice criterion. However, the decisionmakers will use the full range of choice criteria in proportion to the above‐mentioned probabilities.

14 of decision making under risk.

15 those who are not professional game theorists.

16 LC models are specializations of FM models in which the latent variable is categorical. Unless strong restrictive assumptions are imposed, choice criteria do not form a conveniently nested structure within a single general functional form. Instead they form a set with discrete elements having distinct functional forms. Hence, LC models are particularly useful in empirical studies of choice criteria.

17 for example, heterogeneity in risk aversion in EU models, and heterogeneity in probability weighting in PT models.

18 note that standard random utility has no reference point.

19 Although the present formulation moves beyond the simplicity of a single‐decision‐criterion world view, each criterion of Table 1 can be formalized by an existing theoretical framework. Normative deliberation is underpinned by the axiomatizations of, for example, von Neumann and Morgenstern, or Leonard Savage. Descriptively valid, partly deliberative behavioral rationality is underpinned by axiomatizations of Cumulative Prospect Theory by, for example, Wakker and Tversky (1993). The deliberative‐rationality‐displacing role of visceral emotions has been recognized in the evolutionary study of behavior, represented in economics in particular by, for example, Frank (1988). Automaticity, in which deliberative rationality is not so much bypassed as simply “not engaged”, has been given theoretical underpinning in the psychology literature by Moors and DeHouwer (2006). Imperfect and fallible recognition, categorization, and procedural responses have been widely documented (Chou et al., 2009; Frederick, 2005; Goldstein & Taleb, 2007; Kaivanto et al., 2014; VanLehn, 1990), for which general theoretical underpinning may be obtained from, for example, Jehiel's (2005) concept of analogy‐based expectations equilibrium.

20 This is a simplification. It might be that an individual is locked into one particular SoM for an extended period of time—due to,for example, tiredness, stress, or time pressure—and processes all emails in a given session with that one choice criterion. It could also be the case that an individual's SoM evolves through two or more SoM before settling on one choice criterion. In our model, these effects are not modeled explicitly, but the choice‐criterion selection probabilities $\pi_{it}^{c}$ include a time subscript with which such dynamics could be modeled in principle.

21 These are discussed further in Section 4.4.

22 The baseline propensity to adopt a deliberative choice criterion $\chi_{i}^{1}$ is a stable trait (Parker et al., 2017) that can be measured by the cognitive Reflection Test of Frederick (2005), or the Decision‐Making Competence scale of Parker and Fischhoff (2005).

23 We thank an anonymous referee for this insight.

24 Even when a person does process the email through a deliberative process, they may still end up being fooled. However, the probability of clicking through is much smaller than with routine‐automatic processing ($\rho^{4}\gg \rho^{1}$).

25 for example, do not miss out on this “once‐in‐a‐lifetime opportunity!”.

26 for example, law enforcement officers, tax officials.

27 the tendency to repay in kind even though there is no implicit obligation to do so.

28 the tendency to comply with requests made by people whom the user likes or with whom the user shares common interests or common affiliations.

29 Also referred to as “consistency.” People feel obliged to behave in line with—consistently with—their previous actions and commitments.

30 Making an option seem attractive by framing it with respect to an option that is (contrived to be) noticeably less attractive.

31 People conform with majority social opinion, even when this manifestly contradicts immediate personal perception, as in, for example, the Stanford Prison Experiment.

32 The devices and ploys of social engineering (see Sections 2 and 4.2) target aspects of human decisionmakers that are absent in normatively rational decisionmakers.

33 In principle, an organization could substantially reduce ρ^4^ by implementing organizationwide two‐stage security procedures before any email link or attachment is opened, however such measures have not been widely adopted due to their efficiency cost (as discussed in Sections 1 and 2).

34 If logistic regression is used instead, the parameter estimates are log odds ratios, which are harder to interpret.

35 Our results do not conclusively rule out the possibility that *TII* training could outperform the baseline, because we compare here our initial attempts at producing training packages against packages informed and refined over years of industry‐standard approach development by professionals in the field of information security training.

36 “Confidence in the accuracy of intuitive judgment appears to depend in large part on the ease or difficulty with which information comes to mind … and the perceived difficulty of the judgment at hand” (Alter et al., 2007).

## APPENDIX A TRANSCRIPT FOR ROUTINE‐INTERRUPT TREATMENT (*TRI*)

**Slide 1**:
Welcome. This seminar is going to be a little bit different. We cannot supply all the answers; instead we hope to challenge you to consider some important questions.

**Slide 2**:
Let us start with a hard one. Why do so many people fall for phishing attacks?
If you think that you have an easy answer it probably went along the lines of: “because some people are clueless about technology.”
Or, more likely: “because some people are clueless about technology.”
Well, we're at a university so let's look at the evidence…

**Slide 3**:
I have found three studies which examine phishing risk specifically in university staff and students.
The results of all three studies suggest that, even university staff and students, most of whom are fully aware of phishing, still fall victim to its attacks. In fact, around one‐third to one‐half of participants click on simulated phishing links.
But importantly, each of these studies also examines the link between technical awareness and phishing risk. These are their findings:
Dhamija et al. find that participants proved vulnerable across the board to phishing attacks, and that neither their characteristics not their technical experience affected that risk significantly.
Downs et al. also find that technical knowledge is unrelated to phishing risk, regardless of which specific knowledge type they measure.
And Diaz et al. find the unexpected result that greater phishing knowledge actually increases phishing risk.
These findings strongly suggest that we may all be at risk from phishing attacks, but How could we explain this?
Well it could be that those of us who are more technically aware are also more likely to have online accounts, transactions, deliveries and software updates pending, so that phishing scams based upon these subjects are more likely to “ring true” for us.
*It could also be because experience and efficiency in dealing with electronic messages is, itself, our greatest weakness*.

**Slide 4**:
These days, most of us would say that we are very busy. We also tend to receive a high volume of emails.
It is therefore efficient and adaptive for us to process these emails in a fast, almost automatic manner.
But Automatic processing of emails is also very dangerous.
All it takes is one click on a link or attachment and…

**Slide 5**:
Bang!
One click on a bogus link or attachment could lead to my computer, and possibly others across the network, being infected with a virus. Consider: how much would I actually pay to avoid losing all of my files and data. And multiply that cost across the tens of thousands of users at Lancaster.
Let us consider a couple of examples of how hackers can take advantage of automatic processing…

Example 1 Here is a message that most students will be very familiar with. To our automated processing system, it looks precisely like the genuine article, and so we might automatically click on the link to get it done, or possibly flag it to come back to and click‐through this evening. However, this message is a fake. It is not obvious:

- The sender and their address look genuine.
- And, although a generic greeting like “dear student” is often a sign of a phishing scam, in this case the genuine module evaluation email also starts “dear student.”
- Without digging into the message properties there is just one way to recognize that this message is a fake.
- By hovering over the links—without clicking on them—I can see that they do not actually point at lancaster.ac.uk.
- lancasterac.uk is a completely different domain to lancaster.ac.uk—and it could be owned by anyone.

Whenever we are about to click on a link in an email, we need to get into the habit of hovering over that link and looking at the domain section between the double slash and the next slash.
Example 2
(pop‐up window) Sorry about that, it looks like I need to install an update. Ah, one sec, I was just about to automatically click later without checking the link.
adobenotificaitions.co.uk sounds quite legitimate, but it is worth pointing out that it's a totally separate domain to adobe.co.uk/notifications. A quick search can show us that adobe own adobe.com, and we would expect everything they do to operate as a subdomain of that: so adobe.com/uk, or adobe.com/updates , or adobe.com/blahblahblah. In fact I bought adobenotifications.co.uk for £10 so that this example phishing scam that I made could be hosted at that same address.

**Slide 7**:
So to summarize:
  - It is essential to acknowledge that we are all at risk from phishing attacks.
  - If we feel that we are about to click through automatically, we should get into the habit of hovering first.
  - If it is bad, delete it.
  - If you already clicked, contact the service desk immediately: lancaster.ac.uk/ISS/Help

**Slide 8**: So what now?
Well the moodle page that we just came from has a six‐question phishing identification quiz, which we all need to complete right now.
And that quiz includes the opportunity to sign‐up to receive up to three practice emails over the next few months to help keep our skills sharp.
And thank you for listening.

## APPENDIX B TRANSCRIPT FOR IMPULSIVE‐INTERRUPT TREATMENT (*TII*)

**Slide 1**:
Welcome. This seminar is going to be a little bit different. We cannot supply all the answers; instead we hope to challenge you to consider some important questions.

**Slide 2**:
Let us start with a hard one. Why do so many people fall for phishing attacks?
If you think that you have an easy answer it probably went along the lines of: “because some people are clueless about technology.”
Or, more likely: “because some people are clueless about technology.”
Well, we're at a university so let's look at the evidence…

**Slide 3**:
I have found three studies which examine phishing risk specifically in university staff and students.
The results of all three studies suggest that, even university staff and students, most of whom are fully aware of phishing, still fall victim to its attacks. In fact, around one‐third to one‐half of participants click on simulated phishing links.
But importantly, each of these studies also examines the link between technical awareness and phishing risk. These are their findings:
Dhamija et al. find that participants proved vulnerable across the board to phishing attacks, and that neither their characteristics not their technical experience affected that risk significantly.
Downs et al. also find that technical knowledge is unrelated to phishing risk, regardless of which specific knowledge type they measure.
And Diaz et al. find the unexpected result that greater phishing knowledge actually increases phishing risk.
These findings strongly suggest that we may all be at risk from phishing attacks, but How could we explain this?
Well it could be that those of us who are more technically aware are also more likely to have online accounts, transactions, deliveries, and software updates pending, so that phishing scams based upon these subjects are more likely to “ring true” for us.
*But it could also be because our weaknesses do not lie in a lack of ability to recognise phishing scams, but rather in our human psychology*.

**Slide 4**:
Quotations from Sun Tzu, *The Art of War*, c.500 BCE:
  - “If you know the enemy and you know yourself, you need not fear the result of a thousand battles.”
  - “If you know yourself but not the enemy, for every victory gained you will also suffer a defeat.”
  - “If you know neither the enemy nor yourself, you will succumb in every battle.”
Ok, so this research is not all that recent, but it is very relevant. How many of us have paused to really think about how we, ourselves, could be vulnerable to phishing attacks; and how many of us have paused to really think about how attackers could exploit our human vulnerabilities.
We shall do so now.

**Slide 5**:
Sometimes we do not all think as clearly as we would like to. Obvious examples might be when we are tired, intoxicated, angry, or stressed.
In what other states do you think that you, personally, could be less likely to make well‐reasoned decisions?
Phishing attackers exploit our human weaknesses to force poor decision making.
All they need to do is manipulate our emotions so that we make one click and…

**Slide 6**:
Bang!
One click on a bogus link or attachment could lead to my computer, and possibly others across the network, being infected with a virus. Consider: How much would I actually pay to avoid losing all of my files and data? And multiply that cost across the tens of thousands of users at Lancaster.
Let us now consider an example of how hackers can manipulate our emotions:

**Example message**:
Here's a pretty good phishing email.
It doesn't have some of the usual tell‐tale signs:
Its spelling and grammar aren't atrocious;
Its not covered in capital letters and bold font;
It's actually addressed to my name, rather than some generic: “Dear account holder”
(obviously it's not hard to guess this if they have my email address…)
But look at what the message is trying to do: (highlight as reviewed)
The whole message is designed to induce a state of urgency, worry, and guilt. It starts with “reminder.” Have I missed something here?
Contract termination, that sounds serious; overlooked—oh dear; who were these guys anyway—should probably click on the account link to find out;
Debt recovery—that sounds like a major problem—what exactly do they mean—better open the policy to find out…
…And even if I'm confident that I've never heard of these guys, I really should help out the person who's accidentally not receiving these emails by clicking on this sensible‐looking link to let them know…
Obviously, any one click on any of these links or attachments could be disastrous.
We have to develop a higher level of self‐awareness.
We have to consciously notice that this email does an excellent job at provoking a quick, knee‐jerk response that could be extremely damaging.
We all have to notice that and question “Am I being manipulated here?”
Once we ask this question of ourselves, we all have the skills to identify that the email is, in fact, a scam.
We could type the company's name into a search engine and find that it doesn't actually exist, or we could do the same thing with the entirely fictional address or phone number;
Additionally, we could hover our mouse over a link without clicking it, to see that its true address doesn't match up with the in‐text address;
(Btw: identify the host domain by looking at the section after “://” and before the next “/” ),
Ok, time to wrap this seminar up:

**Slide 7**:
So to summarize:
  - “The Enemy” know our vulnerabilities; all they need is one click…
  - We need to acknowledge our own weaknesses, else we can be exploited,
  - We need to listen to our gut, and question anything that tries to target our fears, our desires, or our curiosity … especially if it offers free money.
  - If in doubt, check with a web search, and delete it.

**Slide 8**:
So what now?
Well the moodle page that we just came from has a six‐question phishing identification quiz, which we all need to complete right now.
And that quiz includes the opportunity to sign‐up to receive up to three practice emails over the next few months to help keep our skills sharp.
And thank you for listening.
